# The real-time remote testing and programming of cardiac implantable electronic devices: A case series report

**DOI:** 10.3389/fcvm.2022.1010409

**Published:** 2022-10-13

**Authors:** Yu Long, Shiqiang Xiong, Lin Tong, Jin Li, Yan Luo, Wenchao Huang, Zhen Zhang, Hanxiong Liu, Lin Cai

**Affiliations:** Department of Cardiology, The Third People’s Hospital of Chengdu, Affiliated Hospital of Southwest Jiaotong University, Chengdu Cardiovascular Disease Research Institute, Chengdu, Sichuan, China

**Keywords:** cardiac implantable electronic devices, remote parameter testing, 5G cloud technology support platform, COVID-19, door openings

## Abstract

Minimizing the number of personnel in the cardiac catheterization laboratory (CCL) and the times of CCL door openings contribute to reduce the infection risk of medical staff and patients, particularly during the COVID-19 pandemic. The usage of 5G-CTP system enables device specialists to conduct remote parameter testing and programming without entering the CCL, potentially reducing the exposure risk of medical staff and patients to COVID-19 infection.

## Introduction

The ongoing COVID-19 pandemic has caused extensive devastation throughout the globe and remains the most serious threat to public health since the end of World War II (1), necessitating collective efforts from the entire global population to mitigate the spread of this disease. Owing to their ongoing workplace exposure to the causative virus responsible for this pandemic (SARS-CoV-2), healthcare workers are more likely to suffer from severe disease relative to individuals in other classes of occupations (8.1 vs. 4.1%) (2). This is particularly true for individuals working in operating rooms (ORs), who are particularly likely to suffer from nosocomial infections owing to the large numbers of personnel and mobility in these settings. To minimize the spread of COVID-19 and other diseases, restricting the number of personnel in the OR to the greatest extent possible is of great importance.

Patients preparing to undergo an operation similarly face an increased risk of COVID-19 exposure, and the risk is further amplified for older adults with various comorbidities who are more likely to suffer from severe disease. Frequent OR door openings have also been identified as a risk factor for patients and personnel in an operative setting, and no more than 10 door openings per hour has been used as a surrogate marker for hygiene discipline in the OR (3). When the OR door is frequently opened, this can lead to the disruption of the laminar airflow (4), potentially resulting in surgical site microbial contamination (5, 6), thus exposing patients to a higher risk of surgery-associated infection (7). These OR door openings also have potential impact on surgical teams, further exacerbating the potential for infection or operative errors. As such, counts of OR door openings are often used to gauge the organization and discipline of a given surgical team (8).

In this study, a specific technological approach was used to minimize the number of personnel in a CCL and the associated number of door openings.

## Methods

**FIGURE 1:**
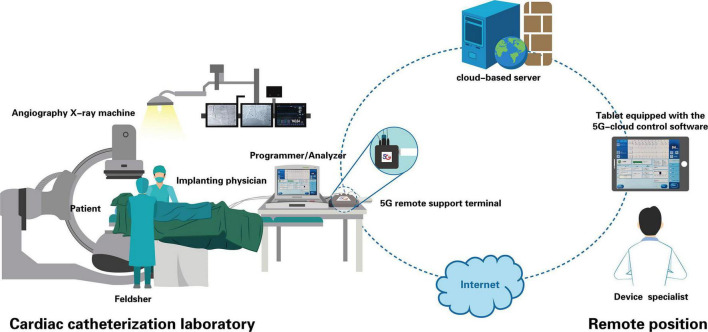
Schematic representation of real-time remote testing and programming of cardiac implantable electronic devices through the 5G-CTP system. This 5G-CTP system is comprised of a 5G remote support terminal, a tablet equipped with the 5G-cloud control software, and a remote service system implemented on a cloud-based server. Prior to device implantation, a feldsher connected externally the 5G remote support terminal to the bedside programmer in the CCL. When confirming the communication is stable, the feldsher got contact with the device specialist. The device specialist logged into the 5G cloud control software subsequently to establish remote connection with the 5G remote support terminal and the programmer. Then he had the complete control of the programmer, and was able to conduct lead parameter testing and programing of implanted device remotely during the implantation procedure to aid implanting physicians in the selection of optimal lead placement. When implantation was complete, final programming parameters were also remotely completed.

For this study, a 5G cloud technology support (5G-CTP) system (China Telecom Corporation Limited Shanghai Branch, Shanghai, China) was utilized that enables real-time remote testing and programming during cardiac implantable electronic device (CIED) implantation procedures over internet connections or wireless networks without requiring entry into the CCL by clinical device specialists (Figure 1). This 5G-CTP system is comprised of a 5G remote support terminal, a tablet equipped with the 5G-cloud control software (China Telecom Corporation Limited Shanghai Branch, Shanghai, China), and a remote service system implemented on a cloud-based server (9–12).

Prior to device implantation, a feldsher or an instrument nurse connected the 5G remote support terminal to the bedside programmer (Merlin Patient Care System Programmer Model 3650, St. Jude Medical Inc., MN, USA) in the CCL, then the device specialist logged into the 5G cloud control software with a two-step verification procedure, as detailed in prior preliminary reports (9–12), thus enabling the establishment of a remote connection with the programmer, providing complete control thereof. During the implantation procedure, the device specialist was able to maintain real-time communication with the implanting physician using an interphone system, and conducted lead parameter testing through the use of the 5G cloud control software to aid implanting physicians in the selection of optimal lead placement while remaining outside the CCL. When implantation was completed, final programming parameters were remotely completed, and the entirety of the remote operative process was recorded through screen recording allowing for subsequent auditing.

For this study, patients were enrolled in an observational trial (ChiCTR2100046883) exploring the feasibility and clinical reliability of this 5G-CTP system in CIED patients. In total, three representative cases that were performed at the Third People’s Hospital of Chengdu, which is a tertiary care center where ∼430 CIED implantation procedures are performed each year, are herein discussed. All patients provided written informed consent to participate, and the ethical review committee of the Third People’s Hospital of Chengdu approved this study ([2021]S-184).

## Results

Case 1: A 76-year-old male suffering from hypertension underwent the implantation of a dual-chamber pacemaker (St. Jude Accent MRI*™* PM2124) for sick sinus syndrome. During the implantation procedure, the 5G-CTP system was used to monitor lead parameters. Device personnel were able to support this implantation process while remaining outside the CCL without the need for frequent door openings. No loss or delay of communication or programmability was observed, and the pacing thresholds, impedance, and sensing thresholds of the atrial and ventricular leads were tested to 1 and 0.6 V, 2 and 21 mV, 503 and 763Ω, respectively. The device was successfully programmed to a DDD of 60 bpm with corresponding lead parameter values within the target range.

Case 2: A 77-year-old male that had been diagnosed with dilated cardiomyopathy with complete left bundle branch block and comorbid diabetes mellitus, hypertension, and chronic cardiac dysfunction, underwent the implantation of a cardiac resynchronization therapy device (CRTP, St. Jude Allure Quadra*™* RFPM3242). During the lead placement procedure, the interphone system was used to enable device personnel to communicate with operators in real-time, while the 5G-CTP system was used to aid in the selection of an optimal position for lead placement. When the pacing thresholds, impedance, and sensing thresholds of the atrial and ventricular leads were tested to 0.6 and 0.75 V, 2.6 and 11.7 mV, and 410 and 360Ω, respectively, the implanting physician fixed the leads. After the procedure was complete, the pacing capture threshold remained stable, and the sensing threshold and lead impedance were within the target range. No adverse interactions with standard CCL care protocols were associated with the use of this 5G-CTP system.

Case 3: An 87-year-old female suffering from atrial fibrillation with rapid ventricular rates was implanted with a dual-chamber pacemaker (Medtronic ADAPTA^®^ L ADDRL1) and underwent atrioventricular junction ablation. The stability of the connection between the 5G-CTP system and the 3650 Merlin analyzer was confirmed by the device specialist. The CCL staff activated the analyzer, while lead parameter testing was performed by the off-site device specialist. When the pacing thresholds, impedance, and sensing thresholds of the atrial and ventricular leads were tested to 0.6 and 0.6 V, 2.1 and 17.1 mV, and 358 and 784Ω, respectively, by an off-sire device specialist, the implanting physician fixed the leads. No pacing inhibition or connectivity interruptions were observed between the 5G-CTP system and the 3650 Merlin analyzer during radiofrequency ablation. Lead measurements were within normal ranges, and final programming was remotely completed successfully.

The baseline characteristics and lead parameter testing outcomes of the enrolled patients are presented in Table 1.

**TABLE 1:** Clinical characteristics of the enrolled patients.

	Case 1	Case 2	Case 3
Age, years	76	77	87
Gender	Men	Men	Women
Body mass index (kg/m^2^)	29.7	19	28.1
LVEF, %	56	46	61
Comorbidity	Hypertension	Diabetes mellitus Hypertension Chronic cardiac dysfunction	Atrial fibrillation
Implant indications	Sick sinus syndrome	DCM with complete LBBB	2nd/3rd degree AV block
Implanted device	Dual-chamber pacemaker	CRT-P	Dual-chamber pacemaker
Device implant	First implantation	First implantation	First implantation
Duration for operation/min	80	312	92
Duration for leads parameter testing/min	25	200	36
Frequency for leads parameter testing/beats	7	17	9
Intraoperative leads parameter testing results			
Atrial leads Pacing thresholds/V thresholds/mV Impedance/Ω	1 2 503	0.6 2.6 410	0.6 2.1 358
Ventricular leads Pacing thresholds/V Sensing thresholds/mV Impedance/Ω	0.6 21 763	0.75 11.7 360	0.6 17.1 784

LVEF, left-ventricular ejection fraction; DCM, dilated cardiomyopathy; LBBB, left bundle branch block; CRT-P, cardiac resynchronization therapy without defibrillator.

## Discussion

Clinical device specialists traditionally provide bedside expertise in the CCL that is specific to the utilized devices in the context of CIED implantation. Traditional CCL care procedures generally require an implanting physician, a feldsher, an instrument nurse, and a clinical device specialist providing bedside expertise in the CCL. However, increasing the number of personnel in the CCL and the number of CCL door openings leave medical personnel and patients at a higher risk of infection, particularly during the COVID-19 pandemic. This risk is particularly pronounced for older adults over 60 years of age, who are more likely to suffer from severe disease (13). Notably, most patients who undergo CIED implantation are in this age group. By utilizing a 5G-CTP system, it is possible for device specialists to conduct real-time zero-contact parameter testing and programming while remaining remotely located, thereby decreasing the number of CCL door openings and the number of personnel in the CCL without compromising patient safety (14). The use of this technology can help minimize the exposure of medical staff and patients to COVID-19. In addition, when providing bedside care, device specialists must wear leaded aprons and thyroid shields. Even with these precautions, they will still suffer from slight radiation exposure. By enabling remote operative procedures, this 5G-CTP system enables real-time device-specific expertise while mitigating both infection- and ionizing radiation-related risks for device specialists.

The utilized 5G-CTP system incorporates robust cybersecurity protocols designed to ensure patient data safety, as detailed in prior preliminary reports (9–12). The medical safety of patients is also the major concern during CIED implantation sessions, and a temporary pacemaker will be implanted before surgery in those patients with complete AV-block or complete sick sinus syndrome with a heart rate < 30 bpm to ensure the patient’s safety. No adverse interactions with temporary pacemakers were associated with the use of this 5G-CTP system. Therefore, the 5G-CTP system can be applied to this subset of patients without adverse events. This technology can be applied for a range of applications such as the remote programming of various devices, including in CIED patients necessitating emergency reprogramming (9), as well as for patients in areas where medical resources are limited and post-implantation follow-up analyses are necessary (11). The potential use of 5G-CTP system in different clinical scenarios indicates the value of this system as a promissing modality for CIED patient management.

The three cases presented herein highlight the efficacy and feasibility of this real-time remote parameter adjustment approach. Since these three initial cases, we have routinely utilized this technology during CIED implantation procedures without any communication problems or adverse events to date. We have also been conducting a prospective randomized controlled study to examine the safety and efficacy of the 5G-CTP system in CIED implantation procedures and to assess its effect on parameter testing time. While in-person interactions with device specialists in the CCL are preferable, this technology is an effective alternative that can be used for the duration of the global COVID-19 pandemic.

## Limitations

This was a single-center observational study that enrolled just three cases. As such, further large-scale prospective randomized controlled studies are necessary to confirm the safety and efficacy of this 5G-CTP system as a tool to aid in CIED implantation procedures. As such, caution is warranted in the application of this technology in the context of CIED implantation pending the results of further clinical research. Although there is currently only one vendor with equipment that can readily function with this 5G-CTP system (St. Jude Medical Inc., Saint Paul, MN, USA), the general concept and service model are applicable to all vendors.

## Conclusion

In summary, this 5G-CTP system can effectively enable the real-time testing and programming of lead parameters in the context of CIED implantation without requiring device specialists to enter into the CCL, thereby reducing the number of CCL door openings, reducing the potential for COVID-19 exposure for both healthcare workers and patients. However, additional large-scale multi-center prospective research will be critical to confirm the utility and safety of this technology.

## Data availability statement

The original contributions presented in this study are included in the article/supplementary material, further inquiries can be directed to the corresponding authors.

## Ethics statement

The studies involving human participants were reviewed and approved by The Third People’s Hospital of Chengdu. The patients/participants provided their written informed consent to participate in this study.

## Author contributions

YuL was the major contributor in drafting the manuscript. SX, LT, JL, YaL, WH, and ZZ put forward constructive comments and suggestions. SX and HL revised the manuscript for important intellectual content. LC designed the study and finally approved the manuscript submitted. All authors have read and approved the final manuscript.
